# Editorial: Agency in posttraumatic stress disorder: theoretical approach and therapeutic perspectives

**DOI:** 10.3389/fnint.2026.1785574

**Published:** 2026-02-02

**Authors:** Vladimir Adrien, Marion Trousselard, Félix Schoeller

**Affiliations:** 1 AP-HP, Avicenne Hospital, Department of Child, Adolescent and General Psychiatry, Bobigny, France; 2 Université Sorbonne Paris Nord, Laboratoire Éducations et Promotion de la Santé (UR 3412), Bobigny, France; 3 Institut de Recherche Biomédicale des Armées, Brétigny-sur-Orge, France; 4 INSPIIRE, Inserm UMR 1319, Université de Lorraine, Nancy, France; 5 Ecole des Psychologues Praticiens, Paris, France; 6 BeSound SAS, Paris, France

**Keywords:** agency, chronesthesia, dissociation, grounding, posttraumatic stress disorder, psychological trauma, psychomotor therapy, sensorimotor

Posttraumatic stress disorder (PTSD) remains a major public health challenge, affecting 3.9% of the world population (Koenen et al., 2017), with only a minority of patients achieving durable remission while many develop chronic, complex, or dissociative forms of PTSD despite evidence-based trauma-focused psychotherapies (Kessler et al., 1995; Steenkamp et al., 2015; Schnurr et al., 2022). Dominant therapeutic models emphasize fear extinction, emotional regulation, and cognitive restructuring. While these approaches are indispensable, they may insufficiently address a more fundamental disruption produced by trauma-related disruption: dissociation, understood as a collapse of grounding to reality, involving alterations in proprioception, interoception, chronesthesia, sense of body ownership and sense of agency. Reconsidering PTSD through the lens of agency offers a unifying framework that connects peritraumatic experience, dissociation, memory fragmentation, and symptom persistence.

## Why agency matters

Agency refers to the prereflective feeling of initiating and controlling one's actions and their consequences (Tsakiris et al., 2007; Haggard and Chambon, 2012). Although conceptually distinct, the sense of body ownership, that refers to the experience that one's body and experiences belong to oneself, and the sense of agency are deeply intertwined within multisensory and predictive processes integrating proprioception, interoception, motor intentions, and sensory feedback. Crucially, agency anchors the self as an *actor* in the world: it is through agency that perception, action, and meaning are dynamically coupled, within the past, present and future. Indeed, acting involves not only controlling one's actions in the present, but also anticipating their future consequences and embedding them within an autobiographical contin.

Psychiatric research has extensively examined alterations of body ownership and control, particularly in dissociation. By contrast, agency has received comparatively little attention in trauma research, despite growing evidence that it is profoundly altered in PTSD and its dissociative subtypes (Rabellino et al., 2018; Ataria, 2015).

## Trauma as a breakdown of agency

From an enactive and predictive-processing perspective, a traumatic event (TE) overwhelms the individual's capacity to act meaningfully on the environment. The TE exceeds available sensorimotor and cognitive models, leaving no reliable predictions to guide perception or action (Friston, 2017; Linson and Friston, 2019). Neurobiologically, this corresponds to hyperactivation of threat-detection systems alongside reduced prefrontal and hippocampal regulation (Shin et al., 2005; Lanius et al., 2010; Akiki et al., 2017).

Clinically, this collapse of agency is experienced as helplessness, freezing, or tonic immobility, hallmark features of peritraumatic distress (Bracha, 2004). These states reflect not merely fear, but a failure of agency—an inability to initiate or modulate action in response to threat. Peritraumatic dissociation may then emerge as a protective response, disconnecting perception and bodily experience from overwhelming stimuli (Rabellino et al., 2018; van der Kolk, 2014). While dissociation can reduce immediate suffering, its intensity predicts subsequent PTSD, suggesting that early agency failure has lasting consequences (Ozer et al., 2003; Brewin et al., 2000).

Trauma thus constitutes more than a disruption of safety or meaning: it is a sensorimotor rupture in which the self is no longer experienced as an embodied and effective agent in the world within a temporal continuity. The traumatic event is not integrated as an episode located in the past, but remains experientally present, fragmented and decontextualized (Rybyk and Medvedev, 2025; Correa et al., 2022; Keidar et al., 2025).

## PTSD symptoms as attempts to restore agency

If trauma entails a collapse of agency, PTSD symptoms can be reinterpreted as efforts—often maladaptive—to restore it. This reframing shifts symptoms from being mere pathological residues to being understandable, though costly, adaptive strategies.

Intrusive thoughts and flashbacks may represent attempts to reprocess the TE and regain control over its unfolding. Repetitive mental replay can be seen as a search for an alternative action trajectory—an effort to reconstruct causality and agency retrospectively (Murray et al., 2022; Shipherd and Salters-Pedneault, 2008; Herman, 1992). Similarly, behavioral reenactments and risk-taking, particularly observed in complex PTSD, may reflect attempts to actively appropriate danger rather than remain passively subjected to it (van der Kolk, 2014).

Avoidance behaviors can be understood as compensatory strategies to reassert agency by controlling exposure to triggers (Mairean, 2020). When internal regulation fails, avoiding environments, sensations, or relationships becomes the only reliable means of preventing further loss of control. Substance use may function analogously, modulating perception and affect to restore a sense of mastery, albeit transiently (Flynn et al., 2022).

Negative cognitions, including guilt and self-blame, paradoxically preserve agency by locating causality within the self rather than in an uncontrollable world. “If it was my fault, then I had control” becomes a psychologically coherent—though damaging—solution to helplessness (Ozer et al., 2003; Keane et al., 2006).

Hypervigilance and chronic arousal further illustrate this logic: constant monitoring of threat aims to prevent future agency collapse, even as it entrenches anxiety and physiological dysregulation (Cameron and Mamon, 2019).

Finally, dissociation occupies a paradoxical position. It simultaneously reflects a loss of agency and an active strategy to counter passive helplessness. By disengaging from perception or embodiment, individuals may reclaim a minimal sense of control over overwhelming experience—at the cost of coherence, presence, and long-term functioning (Morgan, 2013; Taylor and Morgan, 2014).

## Clinical and conceptual implications

Viewing PTSD through the prism of agency has important implications. First, it offers a unifying account linking peritraumatic responses, symptom clusters, and chronicity. Second, it complements cognitive and emotional models by foregrounding the embodied dimension of trauma, emphasizing action, sensorimotor prediction, and environmental engagement. Third, it challenges clinicians to consider not only how patients remember trauma, but how they experience themselves as agents in everyday life, but also within an assimilated past and an anticipable future.

Restoring agency may thus represent a core therapeutic target across PTSD subtypes, including dissociative and complex presentations, for which treatment guidelines remain limited (Brewin et al., 2017; Maercker et al., 2022). While established therapies may indirectly support agency by improving regulation and narrative coherence, an explicit focus on agency could help explain non-response and guide innovation.

## Conclusion

Psychological trauma can be understood as a profound loss of the SA, disrupting the individual's capacity to act meaningfully on the world. PTSD symptoms, in turn, reflect persistent attempts to repair this rupture—attempts that are understandable, adaptive in intent, yet often self-defeating. Re-centering agency in trauma theory invites a shift from viewing PTSD solely as a disorder of fear or memory toward recognizing it as a disorder of action, control, and embodied selfhood. Such a perspective may open new avenues for conceptual clarity and therapeutic progress in a field still marked by significant unmet needs.
